# ADAMTS13 Improves Endothelial Function and Reduces Inflammation in Diabetic Retinopathy

**DOI:** 10.3390/cells14020085

**Published:** 2025-01-09

**Authors:** Ahmed M. Abu El-Asrar, Mohd I. Nawaz, Ajmal Ahmad, Mairaj Siddiquei, Eef Allegaert, Lowie Adyns, Lotte Vanbrabant, Priscilla W. Gikandi, Gert De Hertogh, Sofie Struyf, Ghislain Opdenakker

**Affiliations:** 1 Department of Ophthalmology, College of Medicine, King Saud University, Riyadh 11411, Saudi Arabia; mnawaz@ksu.edu.sa (M.I.N.); aajmal@ksu.edu.sa (A.A.); msiddiquei@ksu.edu.sa (M.S.); pgikandi.c@ksu.edu.sa (P.W.G.); ghislain.opdenakker@kuleuven.be (G.O.); 2 Dr. Nasser Al-Rashid Research Chair in Ophthalmology, College of Medicine, King Saud University, Riyadh 11411, Saudi Arabia; 3 Laboratory of Histochemistry and Cytochemistry, University of Leuven, 3000 Leuven, Belgium; eef.allegaert@kuleuven.be (E.A.); gert.dehertogh@uzleuven.be (G.D.H.); 4 University Hospitals UZ Gasthuisberg, 3000 Leuven, Belgium; 5 Laboratory of Molecular Immunology, Department of Microbiology, Immunology and Transplantation, Rega Institute, University of Leuven, 3000 Leuven, Belgium; lowie.adyns@kuleuven.be (L.A.); lotte.vanbrabant@kuleuven.be (L.V.); sofie.struyf@kuleuven.be (S.S.); 6 Laboratory of Immunobiology, Department of Microbiology, Immunology and Transplantation, Rega Institute, University of Leuven, 3000 Leuven, Belgium

**Keywords:** diabetic retinopathy, ADAMTS13, inflammation, blood–retinal barrier

## Abstract

The protease, a disintegrin and metalloproteinase with thrombospondin type 1 motif member 13 (ADAMTS13), known to cleave only the von Willebrand factor (VWF), has powerful regulatory effects on microvascular platelet adhesion, thrombosis, inflammation, and endothelial dysfunction. We study the protection against diabetes-induced retinal injury in experimental rats by supplementation with recombinant ADAMTS13. We compare human epiretinal membranes and vitreous samples from nondiabetic subjects and patients with proliferative diabetic retinopathy (PDR) and extend in vitro analyses with the use of various immunodetection and spectrofluorimetric methods on rat retina and human retinal glial and endothelial cell cultures. Functional studies include the assessment of the blood–retinal barrier (BRB), cell adhesion, and in vitro angiogenesis. In epiretinal membranes, endothelial cells and monocytes/macrophages express ADAMTS13. The levels of VWF, the platelet marker CD41, ADAMTS13, and the biomarkers of endothelial cell injury soluble VE-cadherin and soluble syndecan-1 are increased in PDR vitreous. ADAMTS13 is downregulated in diabetic rat retinas. The intravitreal administration of ADAMTS13 attenuates diabetes-induced BRB breakdown, the downregulation of VE-cadherin and β-catenin, and the upregulation of VWF, CD41, phospho-ERK1/2, HMGB1, VCAM-1, and ICAM-1. In Müller cells, ADAMTS13 attenuates MCP-1, MMP-9, and ROS upregulation induced by diabetic mimetic conditions. In HRMECs, ADAMTS13 attenuates the shedding of the soluble VE-cadherin and soluble syndecan-1 and the levels of phospho-ERK1/2, MCP-1, fractalkine, and ROS induced by diabetic mimetic conditions, the upregulation of ICAM-1 and VCAM-1 elicited by TNF-α, the adherence of monocytes induced by TNF-α, and VEGF-induced migration of human retinal microvascular endothelial cells. Our findings suggest that enhancing ADAMTS13 levels in situ ameliorates diabetes-induced retinal inflammation and vascular dysfunction.

## 1. Introduction

Microvascular complications of diabetes mellitus include diabetic retinopathy (DR), neuropathy, and nephropathy. The inner blood–retinal barrier (BRB) is composed of endothelial cells that line the microvasculature with their dual basement membrane, surrounded by pericytes and glial cells including astrocytes, Müller cells, and microglia. The overall structure is collectively termed the neurovascular unit. Adjacent endothelial cells of the retina are sealed together by specific junction protein complexes that maintain the integrity of the inner BRB, including tight junctions, adherens junctions, and gap junctions. The breakdown of these tight junctional complexes contributes to the breakdown of the inner BRB and the subsequent retinal edema [1,2]. Chronic low-grade subclinical inflammatory vasculopathy, causing dysfunction of the ocular microvasculature and BRB breakdown, plays a key role in diabetes-induced retinal injury. The involved molecular alterations, i.e., increased expression of retinal intercellular adhesion molecule-1 (ICAM-1) and the leukocyte integrin CD18, may increase the adhesion of circulating leukocytes to the retinal endothelium [3]. Furthermore, the breakdown of the BRB results in enhanced vascular permeability and macular edema in diabetic patients (DME), representing a common cause of impaired vision in patients with diabetes [4]. In addition, platelet-containing microthrombi are detected in the retinal vasculature of diabetic individuals [5] and diabetic rats [6]. These microthrombi can contribute to capillary occlusion and retinal ischemia. In line with these studies, we have previously demonstrated a significant upregulation of the chemokine platelet factor-4 (PF-4/CXCL4) in vitreous samples from patients with proliferative diabetic retinopathy (PDR) and diabetes-induced enhanced PF-4/CXCL4 levels in the retina of rats [7]. PF-4/CXCL4 is a major constituent of platelet α-granules and is released in high amounts upon platelet activation [8].

Despite advances in drug discovery and development, not all patients respond properly to intravitreal anti-vascular endothelial growth factor (VEGF) therapy, which has been adopted as the current treatment for the center involving DME. Moreover, such approaches often lead to transient responses, and a number of patients develop progressive resistance [9]. These findings suggest that DME is regulated by multiple pathways that are able to compensate for each other when a single pathway is inhibited. Therefore, the development and improvement of new therapeutic approaches targeting a range of pathways are highly needed, allowing for more satisfactory disease management and a lower treatment burden.

A disintegrin and metalloproteinase with thrombospondin type 1 motif, member 13 (ADAMTS13) is a protease that belongs to a family of zinc-containing metalloproteinases [10]. In contrast to other ADAMTS family members, ADAMTS13 has a high substrate specificity and is, so far, known to cleave only the von Willebrand factor (VWF), a multimeric protein that mediates platelet adhesion, thrombus formation, and inflammation [10]. Patients with low levels of ADAMTS13 can develop thrombotic thrombocytopenic purpura, characterized by platelet-rich thrombi in the microvasculature [10]. By cleaving the highly reactive ultra-large VWF multimers into smaller, less hemostatically active VWF molecules, ADAMTS13 has been shown to reduce both microvascular thrombosis and inflammation and improve microvascular endothelial dysfunction in experimental models of diabetic nephropathy [11], acute kidney injury [12], myocardial infarction [13], sickle cell disease [14], rheumatoid arthritis [15], stroke [16], and subarachnoid hemorrhage [17]. Thus, the ADAMTS13/VWF pathway has an important role in vascular inflammation and thrombosis. When an increased VWF release outweighs the cleaving capacity of ADAMTS13, an excess of ultra-large VWF multimers is produced, which can induce microthrombi formation that occludes the microcirculation and causes endothelial damage [10]. Previously, we have compared the ocular expression levels of various members of the ADAMTS proteinase family in PDR patients and have documented that ADAMTS13 levels are below those of the other studied ADAMTS members [18]. Based on these observations, we hypothesize that recombinant human (rh) ADAMTS13 might mitigate diabetes-induced retinal injury by improving endothelial dysfunction and inhibiting inflammation.

## 2. Materials and Methods

### 2.1. Patient Samples

A total of 39 patients with advanced proliferative diabetic retinopathy (PDR) donated 0.3 mL to 0.6 mL of vitreous fluid during a planned surgical vitrectomy as a recommended procedure of tractional retinal detachment and/or vitreous hemorrhage. For comparison, vitrectomy samples from a clinical control cohort of 36 patients were analyzed. The latter control patients were in need of retina surgery because of rhegmatogenous retinal detachment due to retinal breaks; however, they did not show any signs of proliferative vitreoretinopathy. During similarly indicated vitrectomies, 14 patients with PDR donated epiretinal fibrovascular membranes that were fixed for 2 h in a 10% formalin solution, embedded in paraffin, and further processed as described [19,20,21].

### 2.2. Immunodetection of ADAMTS13 and Cell Markers in Human Epiretinal Membranes

Various immunohistochemistry protocols, including sequential double immunostaining, were executed as detailed previously [19,21]. Antigen retrieval was performed for CD31 detection by boiling the sections for 10 min in a citrate-based buffer (pH 5.9–6.1), commercially available as Epitope Retrieval Solution 1 from Leica Biosystems, IL, USA, whereas, for CD68 and ADAMTS13 detection, the sections were boiled for 20 min in Tris/EDTA buffer (pH 9) (Epitope Retrieval Solution 2; Leica). Thereafter, we incubated the sections for 1 h with antibodies against CD31 (monoclonal antibody clone JC70A; Dako, Glostrup, Denmark), CD68 (clone KP1; Dako), and ADAMTS13 (rabbit polyclonal antibody 1:400; ab71550 from Abcam, Cambridge, UK). For all antibody preparations, prior titrations were conducted on human spleen, heart, or kidney sections to optimize the staining procedures. For antigen visualization, we incubated the sections for 20 min with enzyme-conjugated IgGs against the respective primary antibodies. Bright red immunoreactive sites were obtained following the alkaline phosphatase enzyme reaction for 15 min with the Fast Red chromogen. Faint counterstaining with commercial Mayer’s hematoxylin from Leica was applied to visualize the microenvironment of the stained cells.

ADAMTS13-producer cell phenotyping was obtained by sequential double immunohistochemistry: to detect monocytes/macrophages, the sections were first incubated with anti-CD68 and then treated with a peroxidase-conjugated secondary antibody and the peroxidase substrate to define these leukocytes. The substrate 3,3′-diaminobenzidine tetrahydrochloride yielded brown precipitates. To detect ADAMTS13-producing cells, the antigen reactivities with the primary antibody against ADAMTS13 were detected as indicated above without application of any counterstain. Control experiments consisted of replacing primary antibodies with a recommended ready-to-use product (DAKO Real antibody Diluent, Code 52022 from Agilent Technologies), which yielded no detectable staining.

### 2.3. Induction of Diabetes in Rats with Streptozoticin

For in vivo evaluation of the effects of ADAMTS13, we used adult male Wistar rats of 8–9 weeks of age (200–220 g) after overnight fasting and the single-bolus high-dose protocol of streptozotocin (STZ), i.e., 60 mg/kg in 10 mM sodium citrate buffer, pH 4.5 (Sigma, St. Louis, MO, USA) injected intraperitoneally. As control we used age-matched rats injected with equal volumes of a citrate buffer. Streptozotocin-mediated pancreatic island β cell death yielded experimental diabetes with blood glucose levels in excess of 250 mg/dL. As detailed elsewhere, rats made diabetic with the use of streptozotocin demonstrate characteristics of nonproliferative diabetic retinopathy that occurs in humans, including increased vascular permeability resulting from the breakdown of the BRB and signs of inflammation. BRB breakdown occurs as early as 2 weeks following treatment with the streptozotocin bolus [22,23]. After 4 weeks of experimentally proven diabetes, retinas were isolated and processed as described [20]. Similarly, retinas were obtained from age-matched nondiabetic control rats.

### 2.4. Intravitreal Injection of ADAMTS13

To evaluate the in vivo effect of ADAMTS13 on the development of diabetic microangiopathy, rats were made diabetic as described above, and, two weeks later, injected with a sterilized solution of rhADAMTS13 (5 μL at 1 ng/μL; Cat No. 4245-AD, R&D Systems, Minneapolis, MA, USA) into the vitreous of the right eye, as documented [19,21]. Left eyes served as controls and received 5 μL of sterile phosphate buffer saline (PBS). The animals were sacrificed 5 days after the intravitreal injections and their retinas were carefully removed.

### 2.5. Analysis of Blood–Retinal Barrier Integrity

Retinas were excised 5 days after the intravitreal injection, and BRB breakdown was evaluated as previously described in detail [19,20]. In short, the breakdown of the BRB enables intravenous fluorescein isothiocyanate (FITC)-conjugated dextran (3–5 kDa, Sigma-Aldrich) to diffuse into tissues within 30 min. To quantify BRB leakage, a blood sample was collected, all rats were perfused with PBS to remove the remaining intravascular FITC, and the retinas were excised, weighed, and homogenized for fluorescence analyses. FITC-conjugated dextran present in each retina was calculated and expressed in µL/(g*h), as previously documented [19,20].

### 2.6. Human Retinal Müller Glial Cell and Human Retinal Microvascular Endothelial Cell Cultures

Human retinal Müller glial cells (MIO-M1) were provided by Prof. A. Limb, Institute of Ophthalmology, University College London, UK, and cultured according to her instructions [19,20,21]. Confluent cell cultures were starved overnight in serum-free DMEM and used for various treatments for 24 h. Human retinal microvascular endothelial cells (HRMECs) were obtained commercially (Cell Systems Corporation, Kirkland, WA, USA) and cultured as reported up to passage 8 for all of our experiments [19,20,21].

The following stimuli were used for the indicated cells or cell lines. Treatment of MIO-M1 or HRMECs with diabetic mimetic conditions was performed in the absence or presence of 1 h pretreatment with ADAMTS13 (100 ng/mL). Diabetic mimetic conditions included a treatment with 300 μM of the hypoxia mimetic agent cobalt chloride (CoCl_2_) (Cat No A1425-L) from Avonchem Limited, 1 ng/mL of recombinant human TNF-α (Cat No 210-TA) from R&D Systems, or 25 mM of glucose (Scharlau, Sentmenat, Spain). For such supernormal glucose (high glucose, HG) treatment of the cells, 25 mM of mannitol (Scharlau) was used as an osmotic control. Cell supernatants were collected after 24 h and the indicated analytes were measured by ELISA.

### 2.7. Analyte Titrations with the Use of ELISAs

Enzyme-linked immunosorbent assay (ELISA) kits for human vascular endothelial growth factor (VEGF) (Cat No DY293B), human monocyte chemotactic protein-1 (MCP-1)/CCL2 (Cat No DY279), human soluble syndecan-1 (Cat No DY2780), human matrix metalloproteinase-9 (MMP-9) (Cat No DY911), and human soluble VE-cadherin (Cat No DCADV0) were purchased from R&D Systems. An ELISA kit for human fractalkine/CX3CL1 (Cat No ab192145) was purchased from Abcam.

Levels of human soluble VE-cadherin and soluble syndecan-1 in vitreous fluid and VEGF, MCP-1, MMP-9, syndecan-1, and soluble VE-cadherin in the culture medium were determined with the aforementioned ELISA kits according to the manufacturer’s instructions. The minimum detection limits for soluble syndecan-1, soluble sVE-cadherin, VEGF, MCP-1, MMP-9, and fractalkine ELISA kits were approximately 50 pg/mL, 113 pg/mL, 12 pg/mL, 9 pg/mL, 10 pg/mL, 1.18 pg/mL, respectively.

### 2.8. Analysis of Human Vitreous Fluid, Human Retinal Microvascular Endothelial Cell, and Rat Retina Lysates by Specific Immunoblots

We lysed retina and cell samples in 30 mM of Tris-HCl, pH 7.5 buffer, containing 5 mM of EDTA, 250 mM of sucrose, 1 mM of sodium vanadate, 1% Triton X-100, and the protease inhibitor cocktail Complete (Roche, Mannheim, Germany). Tissue and cell homogenates were centrifuged (14,000× *g* for 15 min, 4 °C), and protein concentrations were determined in the supernatant fluids. Protein extracts (30 or 50 μg) were electrophoresed in SDS-containing polyacrylamide gels and the separated proteins transferred, as described previously [19,20,21].

To determine the presence of VWF, CD41, and ADAMTS13 in the vitreous samples, 15 μL aliquots of vitreous samples were boiled for 10 min in Laemmli’s sample buffer (1:1, *v*/*v*) under reducing conditions. After initial testing with the commercial antibodies against ADAMTS13 from Abcam and Novus Biologicals, LLC, Centennial, USA, we tested three mouse monoclonal anti-ADAMTS13 antibodies 3H9 (0.75 µg/mL), 12H6 (0.75 µg/mL) and 5C11 (0.75 µg/mL) (developed in the laboratory of Professor K. Vanhoorelbeke at KULAK/KU Leuven, Belgium). These antibodies were tested under non-reducing conditions. The 3H9 antibody recognizes the N-terminus, i.e., the metalloprotease domain of ADAMTS13, the 5C11 recognizes the middle part of the thrombospondin type 1 repeat (TSR2) domain of ADAMTS13, and the 12H6 antibody recognizes the C-terminus, i.e., CUB2 domain of ADAMTS13 [24,25].

The immunodetection of specific molecules was conducted with the following reagents and conditions: VWF with a mouse monoclonal anti-VWF antibody (1:1000, sc-365712, Santa Cruz Biotechnology Inc., Santa Cruz, CA, USA), CD41 with a mouse monoclonal anti-CD41 antibody (1:1000, sc-365938, Santa Cruz Biotechnology Inc.), β-catenin with a goat polyclonal anti-ß-catenin antibody (1:1000, AF1329, R&D system, Minneapolis, MN, USA), ADAMTS13 with a rabbit monoclonal anti-ADAMTS13 antibody (1:1000, NBP3-16038, Novus Biologicals, Littleton, CO, USA) and with the three mouse monoclonal antibodies 3H9, 5C11, and 12H6, as described above, HMGB1 with a rabbit polyclonal anti-high mobility group box-1 (HMGB1) (1:1000, Cat. no. ab18256, Abcam, Cambridge, UK), ERK1/2 with a rabbit monoclonal anti-phospho-extracellular signal-regulated kinase (ERK)1/2 antibody (1:1000, MAB1018, R&D Systems), ICAM-1 with a mouse monoclonal anti-intercellular adhesion molecule-1 (ICAM-1) antibody (1:100, sc-8439, Santa Cruz Biotechnology Inc.), and VCAM-1 with a mouse monoclonal anti-vascular cell adhesion-1 (VCAM-1) antibody (1:100, sc-13160, Santa Cruz Biotechnology Inc.).

The nitrocellulose membranes were treated with 5% non-fat milk made in Tris-buffered saline containing 0.1% Tween-20 (TBS-T) for 90 min at room temperatures, followed by three washings with TBS-T washings (5 min each). Thereafter, the secondary antibodies were added for 1 h at room temperature. The following secondary antibodies were used: goat anti-rabbit immunoglobulin (SC-2004), goat anti-mouse immunoglobulin (SC-2005) (1:100,000, Santa Cruz Biotechnology Inc.), and goat anti-mouse immunoglobulin (1:10,000, HAF007, R&D Systems). To control for equal protein loading in each electrophoresis lane, membranes were stripped and reprobed either with the β-actin-specific antibody (1:2000, sc-47778, Santa Cruz Biotechnology Inc.) or the β-tubulin-specific antibody (1:2000, ab21058, Abcam). Individual protein immunostainings were visualized through the use of high-performance chemiluminescence (G: Box Chemi-XX9 from Syngene, Synoptic Ltd., Cambridge, UK), and the band intensities were quantified with the use of the GeneTools software file version: 4.3.17.0 (Syngene by Synoptic Ltd.).

### 2.9. Cell Adhesion Assay

Adhesion of leukocytes to monolayers of stimulated HRMECs was assayed with a commercial system (CytoSelect Leukocyte-endothelium adhesion kit, Cat. No. CBA-210, Cell Biolabs, Inc., San Diego, CA, USA), as documented in previous work [19,20,21]. Confluent monolayers of overnight starved endothelial cells were pretreated with or without 100 ng/mL of ADAMTS13 and stimulated with 1 ng/mL of TNF-α. The addition of fluorescent-labelled monocytic THP-1 cells to the treated HRMECs monolayer, the washing of non-adherent cells, and the measurement of fluorescence from adherent cells were conducted as described previously [19,20,21].

### 2.10. Measurement of Reactive Oxygen Species

Reactive oxygen species (ROS) generation was measured in Müller glial cells or HRMEC monolayers using a 2′-7′-dichlorofluorescein-diacetate (DCFH-DA). Briefly, cells were grown in standard cell culture media so that 3 × 10^6^–4 × 10^6^ cells were obtained the day before the experiment. Next, the cells were harvested and seeded in a dark, clear bottom 24-well microplate with 1 × 10^5^ cells per well. The cells were allowed to adhere overnight. After washing with 250 μL/well of PBS, the cells were stained by adding 200 μL/well of the 5 μM DCFH-DA (Invitrogen, Waltham, MA, USA) for 45 min at 37 °C in the dark. After removing the DCFH-DA solution, cell monolayers were washed with PBS and were treated either with 10 mM of hydrogen peroxide (H_2_O_2_) (Scharlau, Barcelona, Spain) for 10, 20, 40, 60, 80, 100, and 120 min or with 25 mM of glucose for 1, 12, 24, 36, and 48 h. Following the analyses of such time courses, we pretreated the cells at the highest ROS generation time point with a medium or ADAMTS13 (100 ng/mL) for 1 h. For the high glucose treatment, 25 mM mannitol (Scharlau) was used as a control. Cellular ROS production was measured immediately by fluorescence analysis (excitation and emission wavelengths of 488 nm and 525 nm, respectively) on a SpectraMax Gemini-XPS apparatus from Molecular Devices. To normalize the fluorescence intensities with protein concentrations, the cells were lysed and 1 μL of the supernatant transferred to a clear 96-well plate containing 100 μL of 1:5 diluted protein assay (Bradford assay) solution to measure the protein concentration.

### 2.11. In Vitro Migration Assays

HRMECs were seeded at 1 × 10^5^ cells/well on six-well culture plates and allowed to grow until an 80–90% confluency. The use of a minimal medium was intended to induce quiescence. Scratches were made in the cell monolayers with sterile pipette tips and then the cells were rinsed with PBS and left untreated or treated with 10 ng/mL of recombinant VEGF in the absence or presence of 60 ng/mL or 200 ng/mL or 600 ng/mL of recombinant ADAMTS13 for 16 h. Cell migration was recorded microscopically and analyzed with the use of the Image J software (latest v. 1.54m), as described previously [20].

Alternatively, HRMEC migration across a membrane fused to gold microelectrodes was assessed with the use of cell invasion/migration (CIM) plates and an xCELLigence apparatus (both from Agilent), as described previously [20]. Before the addition to the chemotaxis plate, the cells were pretreated for 30 min with 6 to 600 ng/mL of ADAMTS13. The chemotactic activity was monitored during 20 h and expressed as cell index. This latter parameter increased as the cells migrated across the membrane. Each experiment was performed in duplicates. The obtained cell indices after 7 h (time point where the maximal index was reached) were normalized to the cell index of unstimulated endothelial cells (set at 100%).

### 2.12. Statistical Analysis

Data were analyzed and figures prepared using SPSS^®^ version 21.0 (IBM Inc., Chicago, IL, USA). To test for normality, the Shapiro–Wilk test and Q-Q plots were used. Consequently, the normally distributed data were presented as means ± SD (standard deviation) and range and illustrated using bar charts showing the standard deviations. Alternatively, data were presented as medians (IQR) (interquartile range) and box and whisker plots prepared. One-way ANOVA and independent t-tests were used for the normally distributed data, while Kruskal–Wallis and Mann–Whitney U tests were used for the data that were not normally distributed (adjusted using the Bonferroni correction). Statistical significance was reported when the value was below 0.05.

## 3. Results

### 3.1. PDR Patients Express ADAMTS13 in Epiretinal Fibrovascular Membranes

**Figure 1:**
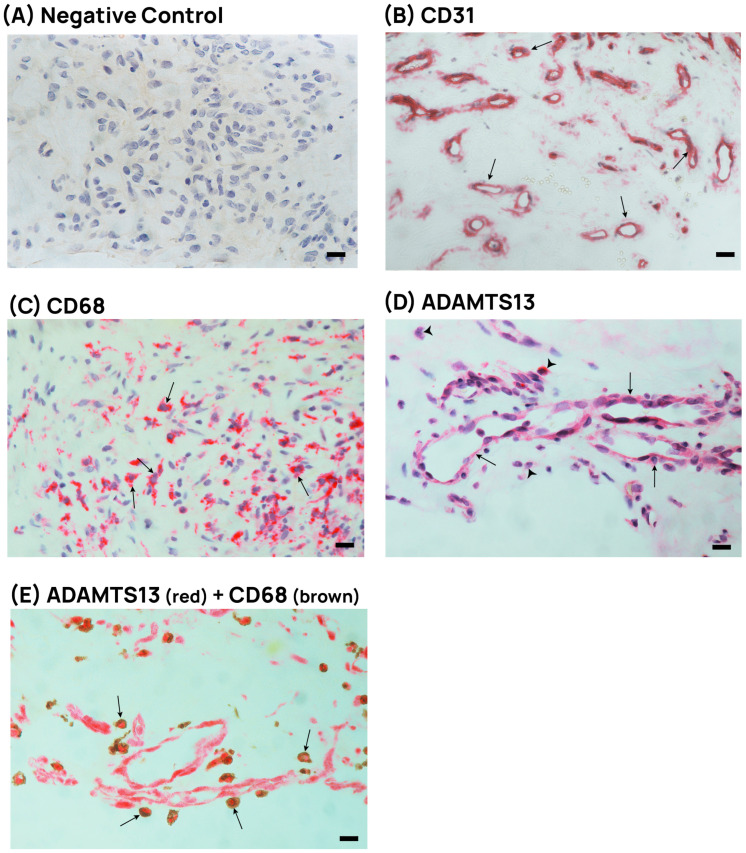
Epiretinal membranes from PDR patients contain endothelial and stromal cells expressing ADAMTS13. (**A**) Negative control slide showing no staining. (**B**) Staining for the endothelial cell marker CD31 showing new blood vessels (arrows). (**C**) Staining for CD68 identifying monocytes/macrophages in the stroma (arrows). (**D**) Staining for ADAMTS13 showing immunoreactivity in vascular endothelial cells (arrows) and in stromal cells (arrowheads). (**E**) Double immunohistochemical staining for ADAMTS13 (red) and CD68 (brown) showing co-expression in stromal cells (arrows). No counterstain to visualize the cell nuclei was applied (black scale bar, 10 µM).

Epiretinal membranes from patients with PDR (*n* = 14) were studied by immunohistochemistry to examine the tissue localization and expression of ADAMTS13. No staining was observed in the negative control slides (Figure 1A). All membranes showed neo-vessels that were positive for the vascular endothelial cell marker CD31 (Figure 1B). Monocytes/macrophages expressing CD68 (Figure 1C) were detected in all membranes. Immunoreactivity for ADAMTS13 was detected in endothelial cells lining new blood vessels in only five samples (Figure 1D). Immunoreactivity for ADAMTS13 was also observed in stromal cells in all membranes (Figure 1D). Co-localization studies revealed that ADAMTS13 immunoreactivity was detected in stromal monocytes/macrophages expressing CD68 in all samples (Figure 1E).

### 3.2. Levels of von Willebrand Factor, the Platelet Marker CD41, and ADAMTS13 in Vitreous Samples

**Figure 2:**
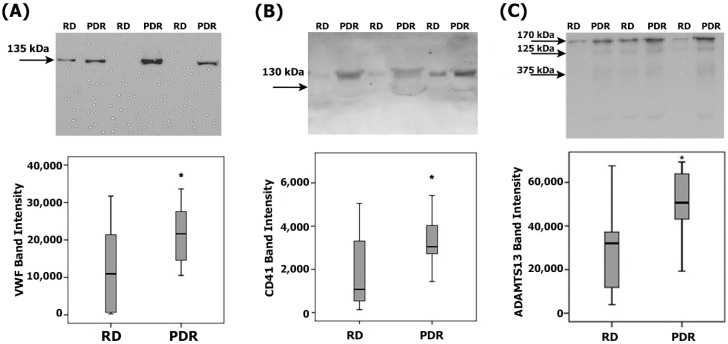
Hemostasis biomarkers in vitreous fluid of diabetes patients. Detection of thrombotic markers in vitreous fluid of patients with proliferative diabetic retinopathy (PDR). Determination of the von Willebrand factor (VWF) (**A**), the platelet marker CD41 (**B**), and ADAMTS13 (**C**) levels in vitreous fluid samples. A total of 15 µL of vitreous fluid samples from 12 patients with PDR and from 12 nondiabetic patients with rhegmatogenous retinal detachment (RD) was subjected to gel electrophoresis and the presence of VWF, CD41, and ADAMTS13 (5C11 monoclonal antibody) was illustrated by representative western blots and the levels of the antigens compared between the RD and PDR cohorts. Results are expressed as medians (interquartile range). (* *p* < 0.05; Mann-Whitney test).

The presence of VWF (Figure 2A), the platelet marker CD41 (Figure 2B), and ADAMTS13 (Figure 2C) was evaluated in identical aliquots of vitreous fluid with the use of the western blot. In comparison with ELISA, which provides single numbers, this type of analysis provides information about identities and relative abundancies of all immunoreactive proteoforms. ADAMTS13 immunoreactivities were expressed as three protein bands at approximately 170 kDa, 125 kDa, and 75 kDa. The predominant ADAMTS13 proteoform was found at 170 kDa, corresponding to the full-length ADAMTS13, whereas the lower molecular weight immunoreactive proteoforms might represent proteolytically truncated ADAMTS13 [26]. In Figure 2C, ADAMTS13 immunodetection was performed with the use of the 5C11 monoclonal antibody. With the 12H6 and 3H9 monoclonal antibodies and the commercial antibodies from Abcam and Novus Biologicals, we detected similar immunoreactivities. Scanning analysis indicated increased levels of VWF, CD41, and ADAMTS13 in PDR vitreous in comparison with the controls.

### 3.3. Levels of Biomarkers of Endothelial Cell Injury and Dysfunction in Vitreous Samples

**Figure 3:**
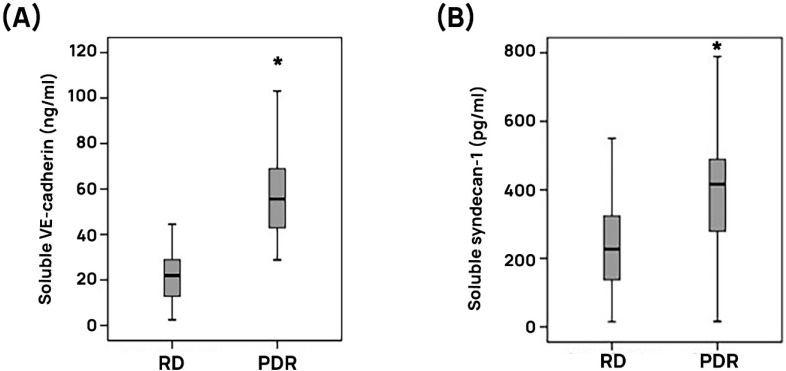
Markers of endothelial injury and dysfunction are enhanced in the vitreous fluid of patients with proliferative diabetic retinopathy (PDR). Soluble vascular endothelial (VE)-cadherin (**A**) and soluble syndecan-1 (**B**) levels were quantified by ELISA and expressed as medians (interquartile ranges) in equal aliquots of vitreous fluid derived from 39 PDR patients and 36 nondiabetic patients with rhegmatogenous retinal detachment (RD) (* *p* < 0.05; Mann–Whitney U test).

We used ELISA to compare the vitreous levels of soluble VE-cadherin and soluble syndecan-1 in 39 PDR patients and 36 nondiabetic controls. Both analytes were detected in all vitreous samples. The median (IQR) level of soluble VE-cadherin in vitreous samples from patients with PDR was 55.6 (42.5–69.1) ng/mL. The median (IQR) concentration in the nondiabetic controls was 22.0 (12.9–30.2) ng/mL. PDR patients had significantly higher levels than nondiabetic controls (*p* < 0.001; Mann–Whitney U test) (Figure 3A). The median (IQR) level of soluble syndecan-1 in PDR patients was 416.6 (268.7–522.0) pg/mL and in nondiabetic controls was 226.8 (134.5–323.8) pg/mL (*p* < 0.001; Mann–Whitney U test) (Figure 3B).

### 3.4. Retinal Expression of ADAMTS13 in an Experimental Rat Model of Diabetes

**Figure 4:**
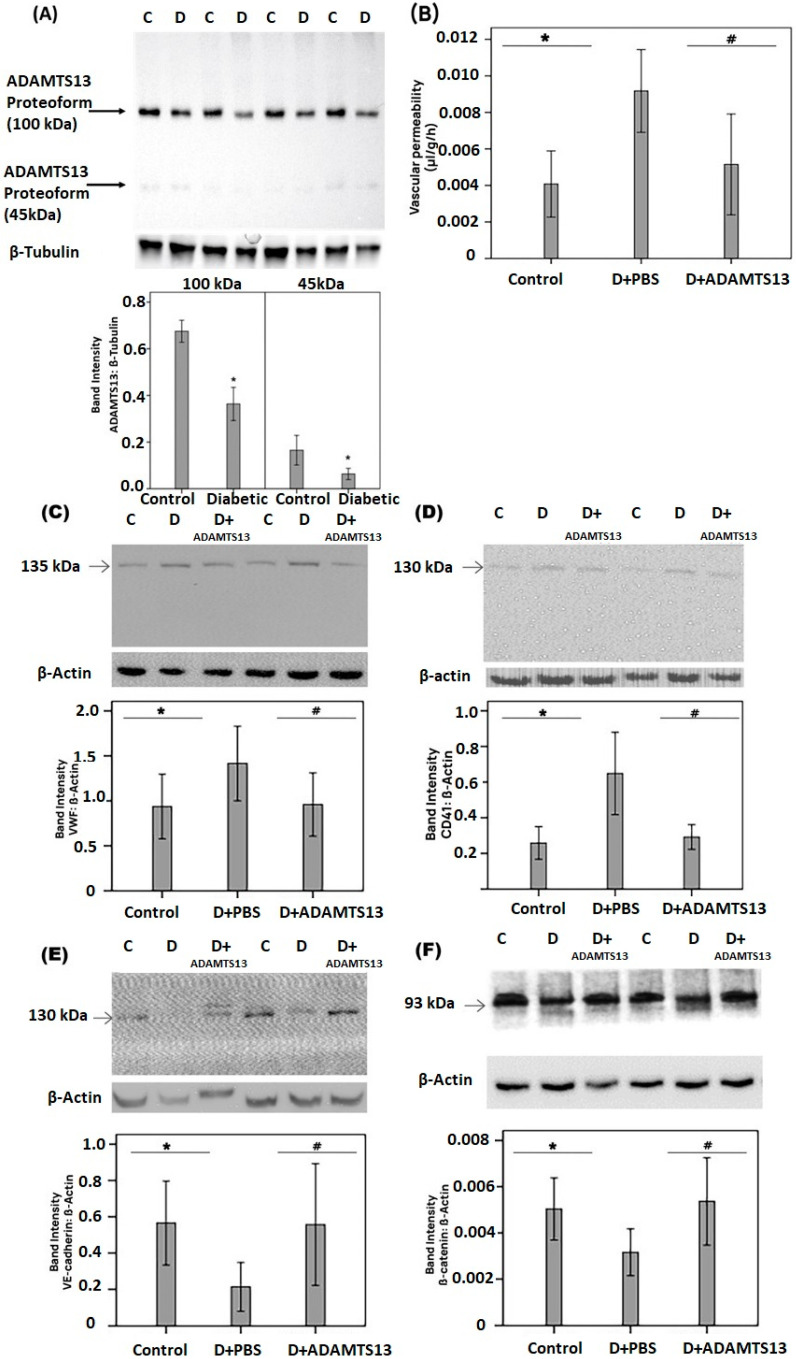
ADAMTS13 expression levels in diabetic rat retinas and effects of exogenously administered ADAMTS13. ADAMTS13 expression levels in the retinal lysates of diabetic rats (**D**) (*n* = 12) and nondiabetic control animals (*n* = 12) were determined by western blot analysis. After the measurement of the intensities of ADAMTS13 proteoform bands, the immunoblots were stripped and reprobed to evaluate ß-tubulin intensities in all sample panels (**A**). Results are expressed as means ± standard deviation of the ratios between ADAMTS13 and ß-tubulin (* *p* < 0.05; independent t-test). The effects of intravitreal ADAMTS13 injection on vascular permeability and markers of hemostasis and inflammation in rat retinas after streptozotocin-induced diabetes were evaluated by quantifications of the BRB breakdown by detection of FITC dextran seeped into the retina after the systemic injection (**B**). Retinal protein expression levels of the von Willebrand factor (VWF) (**C**), the platelet marker CD41 (**D**), vascular endothelial (VE)-cadherin (**E**), and ß-catenin (**F**) were determined by immunoblot analysis. Statistical comparisons (mean ± standard deviation of 8–10 rats) were performed as described in Section 2.12. * *p* < 0.05 compared with values obtained from nondiabetic controls. # *p* < 0.05 compared with values obtained from diabetic rats.

Regulation of retinal ADAMTS13 expression was also studied in a rat model of diabetic retinopathy. With the use of western blot analysis, by applying the commercial antibody from Novus Biologicals, we detected ADAMTS13 as two protein bands at approximately 100 kDa and 45 kDa. The predominant ADAMTS13 proteoform was found at 100 kDa. Densitometric analyses demonstrated decreased ADAMTS13 protein levels in the retina of rats after 4 weeks of STZ-induced diabetes (Figure 4A).

### 3.5. Effect of Intravitreal Administration of ADAMTS13 on Blood–Retinal Barrier in Diabetic Rats

As diabetic conditions in rats were associated with decreased levels of retinal expression of ADAMTS13, we hypothesized that enhancing the expression of ADAMTS13 might ameliorate diabetes-induced retinal injury. Therefore, we used injection of exogenous ADAMTS13 to rat eyes and analyzed various biological and biochemical parameters of retinal integrity and functions. Fluorescein isothiocyanate-conjugated dextran was used to determine the extent of the breakdown of the BRB. In STZ-diabetic rats, retinal vascular permeability was significantly increased compared with nondiabetic rats. Treatment with intravitreal ADAMTS13 significantly reduced the diabetes-induced breakdown of the BRB compared with PBS-treated diabetic eyes (Figure 4B).

### 3.6. Effect of Intravitreal Administration of ADAMTS13 on Retinal Platelet Recruitment, Expression of the Adherens Junction Proteins, and Inflammation in Diabetic Rats

**Figure 5:**
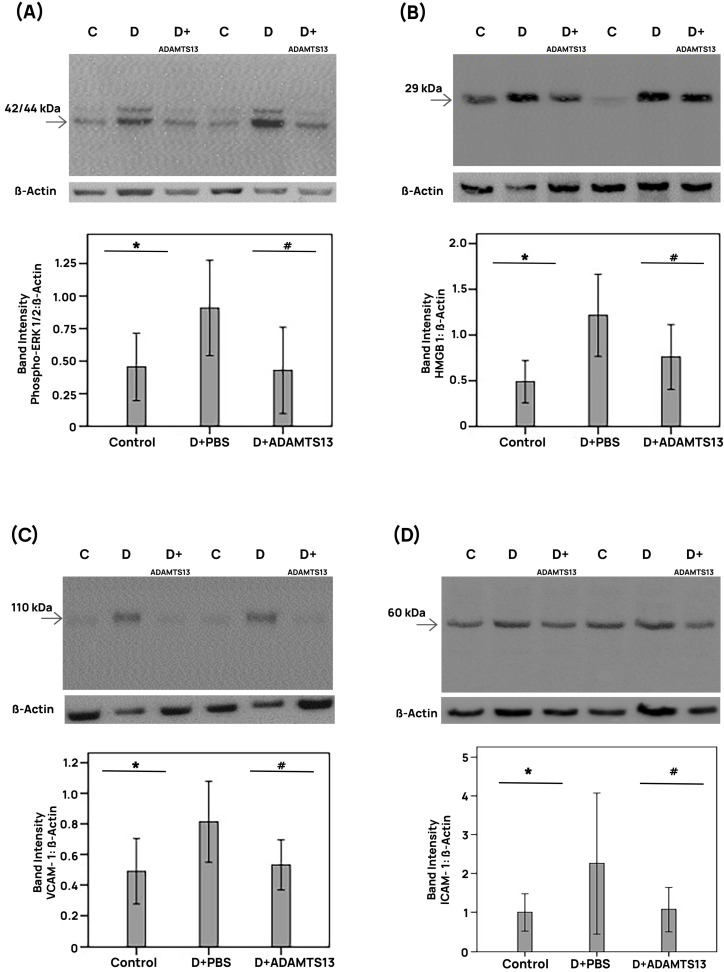
Intravitreal administration of ADAMTS13 reduces retinal inflammation in diabetic rats. The relative protein amounts of phospho-ERK1/2 (**A**), high-mobility group box-1 (HMGB1) (**B**), vascular cell adhesion molecule-1 (VCAM-1) (**C**), and intercellular adhesion molecule-1 (ICAM-1) (**D**) were determined in rat retinas with the use of western blots. The animals were made diabetic with the use of a single streptozotocin bolus, ADAMTS13 was injected in the vitreous, and its effects on inflammation markers were evaluated by comparison of ADAMTS13-injected with the contralateral PBS-injected eyes in single animals. Statistical comparisons (mean standard deviation of 8–10 rats in each group) were performed as described in Section 2.12. * *p* < 0.05 compared with nondiabetic controls. # *p* < 0.05 compared with diabetic rats.

STZ treatment significantly increased the retinal levels of VWF (Figure 4C) and of the platelet marker CD41 (Figure 4D) compared with the retinas of nondiabetic control rats. Treatment with intravitreal ADAMTS13 significantly reduced the levels of retinal VWF (Figure 4C) and CD41 (Figure 4D) in STZ-induced diabetic rats compared with the values obtained from the contralateral diabetic eye that received PBS. Diabetes significantly reduced the retinal protein levels of the adherens injunction proteins VE-cadherin and ß-catenin compared with the retinas of nondiabetic rats. Treatment with intravitreal ADAMTS13 significantly increased the expression of retinal VE-cadherin (Figure 4E) and ß-catenin (Figure 4F) in comparison with the values obtained from the contralateral diabetic eye that received PBS alone. Diabetes significantly increased the retinal expression of phospho-ERK1/2, HMGB1, VCAM-1, and ICAM-1 in comparison with the retinal levels in nondiabetic control rats. Treatment with intravitreal ADAMTS13 significantly reduced the expression of retinal phospho-ERK1/2 (Figure 5A), HMGB1 (Figure 5B), VCAM-1 (Figure 5C), and ICAM-1 (Figure 5D) proteins in STZ-induced diabetic rats in comparison with the levels in the PBS-treated contralateral diabetic eyes.

### 3.7. Anti-Angiogenic and Anti-Inflammatory Effects of ADAMTS13 on Cultured Human Retinal Müller Glial Cells

**Figure 6:**
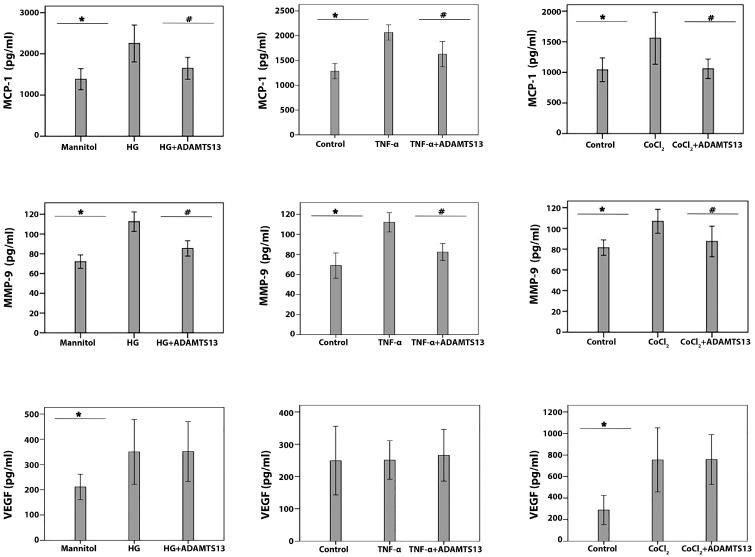
Regulation of proangiogenic and inflammatory molecule expression by ADAMTS13 in human retinal Müller glial cells. Human glial cells were left untreated or treated with high glucose (HG) (25 mM), tumor necrosis factor-α (TNF-α) (1 ng/mL), or cobalt chloride (CoCl_2_) (300 µM) for 24 h or ADAMTS13 (100 ng/mL) for 1h followed by HG, CoCl_2_, or TNF-α. A total of 25 mM of mannitol was used as an inert control for osmotic effects by HG treatment. Levels of monocyte chemotactic protein-1 (MCP-1), matrix metalloproteinase-9 (MMP-9), and vascular endothelial growth factor (VEGF) were quantified in the culture media by ELISA. The present data were generated from three different experiments, each performed in triplicates, and the results are provided as means ± standard deviation; statistical comparisons were performed as described in Section 2.12. * *p* < 0.05 indicates the comparisons with values obtained from control cells. # *p* < 0.05 documents the differences with values obtained from cells treated with HG, TNF-α, or CoCl_2_.

Following the demonstration that ADAMTS13 levels were decreased under diabetic conditions in the retinas of rats and that administration of exogenous ADAMTS13 had beneficial effects on various parameters of retinal integrity in vivo, we started to dissect the effects of ADAMTS13 on diabetic conditions at the cellular level, in vitro. The diabetic mimetic conditions HG, the proinflammatory cytokine TNF-α, and the hypoxia mimetic agent CoCl_2_ significantly induced MMP-9 and MCP-1/CCL2 levels in the culture medium of Müller glial cells compared to untreated control, as measured with the use of specific ELISAs. In Müller cells, ADAMTS13 pretreatment significantly attenuated MCP-1/CCL2 (Figure 6) and MMP-9 levels (Figure 6), which were induced by HG, TNF-α, or CoCl_2_. HG and CoCl_2,_ but not TNF-α, significantly upregulated VEGF levels. Pretreatment of Müller cells with ADAMTS13 did not affect the HG- and CoCl_2_-induced upregulation of VEGF. Müller cells were poor producers of fractalkine/CX3CL1, as this proinflammatory chemokine could not be detected in the supernatants of unstimulated or TNF-α-treated cells.

### 3.8. Cultered Microvascular Endothelial Cells from Human Retina Do Not Express ADAMTS13

By analyzing cell lysates, we tried to demonstrate ADAMTS13 expression in HRMECs. However, we were not able to detect ADAMTS13 expression in these experiments with the commercial antibodies against ADAMTS13 from Abcam and Novus Biologicals, nor with the monoclonal antibodies 3H9, 12H6, and 5C11 against human ADAMTS13.

### 3.9. Protective Effect of ADAMTS13 on Endothelial Cell Dysfunction Induced by Diabetic Retinopathy-Associated Mechanisms

**Figure 7:**
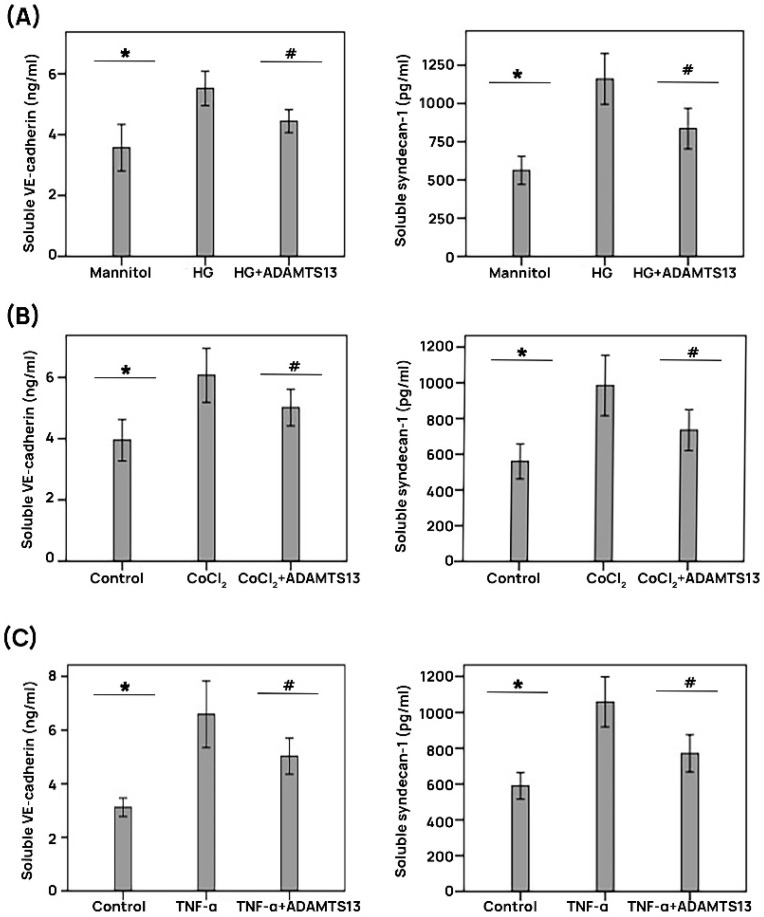
ADAMTS13 promotes the integrity of adherens junctions in human retinal microvascular endothelial cells. Retinal endothelial cells were left untreated (control) or were cultured in the presence of 25 mM of high glucose (HG) concentrations (**A**), 300 µM of cobalt chloride (CoCl_2_) (**B**), or 1 ng/mL of TNF-α (**C**) for 24 h. A third type of treatment consisted of the pretreatment with 100 ng/mL of ADAMTS13 for 1 h followed by HG, CoCl_2_, or TNF-α. A total of 25 mM of mannitol was used as a control for the treatment with high glucose. Levels of soluble VE-cadherin (left histograms) and soluble syndecan-1 (right histograms) in cell culture media were quantified with the use of specific ELISAs. The data represent means ± standard deviations from different (*n* = 3) experiments performed in triplicates, and statistical comparisons were performed as described in Section 2.12. * *p* < 0.05 indicates comparisons with values obtained from control cells. # *p* < 0.05 provides the comparisons with values obtained from cells treated with HG, CoCl_2_, or TNF-α.

ELISA analysis demonstrated that treatment of HRMECs with HG, CoCL_2_, and TNF-α induced significant upregulation of soluble VE-cadherin and soluble syndecan-1 in the culture medium compared to the untreated control. Pretreatment with ADAMTS13 significantly attenuated the levels of soluble VE-cadherin and soluble syndecan-1 induced by HG, CoCl_2_, and TNF-α (Figure 7A–C).

### 3.10. Effect of ADAMTS13 on Inflammatory Signaling Pathways and Inflammatory Marker Expression in Human Retinal Microvascular Endothelial Cells

**Figure 8:**
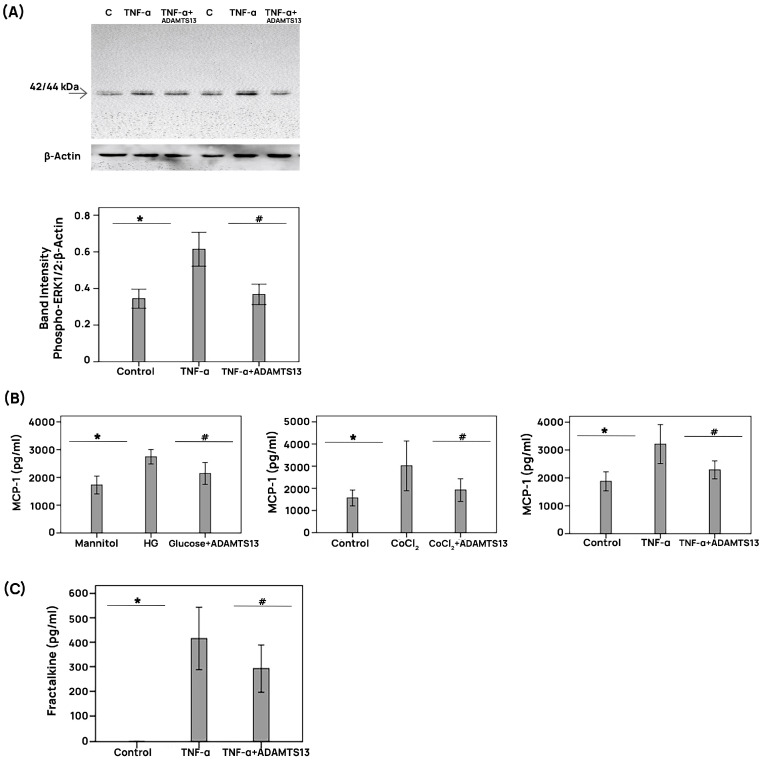
Regulation of inflammatory cytokine expression by ADAMTS13 in human retinal microvascular endothelial cells. Endothelial cultures were left untreated (control) or were treated with 25 mM of high glucose (HG), 300 µM of cobalt chloride (CoCl_2_), or 1 ng/mL of tumor necrosis factor-α (TNF-α) for 24 h with or without a 1 h preincubation with 100 ng/mL of ADAMTS13. A total of 25 mM of mannitol was used as control for cell damage induced by high glucose levels. In panel (**A**), signaling events were probed by measurement of phospho-ERK1/2 levels in cell lysates. In panel (**B**), cell culture medium levels of monocyte chemotactic protein-1 (MCP-1) and, in panel (**C**), cell culture medium levels of fractalkine were quantified with the use of specific ELISAs. Data are expressed as means ± standard deviation from independent (*n* = 3) experiments with triplicates per experiment. Statistical comparisons were performed as described in Section 2.12. * *p* < 0.05 indicates comparisons with values obtained from control cells. # *p* < 0.05 indicates comparisons with values obtained from stimulated cells.

Treatment of HRMECs with TNF-α induced significant upregulation of phospho-ERK1/2 protein levels. Pretreatment with ADAMTS13 significantly attenuated this TNF-α-induced phospho-ERK1/2 increase (Figure 8A). ELISA analyses revealed that the treatment of HRMECs with HG, CoCl_2_, and TNF-α induced significant upregulation of MCP-1/CCL2 in the culture medium versus the control. Pretreatment of HRMECs with ADAMTS13 significantly attenuated the levels of MCP-1/CCL2 induced by HG, CoCl_2_, and TNF-α (Figure 8B). Resting HRMECs did not release detectable amounts of fractalkine/CX3CL1; however, fractalkine/CX3CL1 expression increased above the detection limit in the presence of TNF-α. Pretreatment with ADAMTS13 significantly attenuated the levels of fractalkine/CX3CL1 upregulated by TNF-α (Figure 8C).

### 3.11. Effect of ADAMTS13 on THP-1 Monocyte Adhesion to Human Retinal Microvascular Endothelial Cells

**Figure 9:**
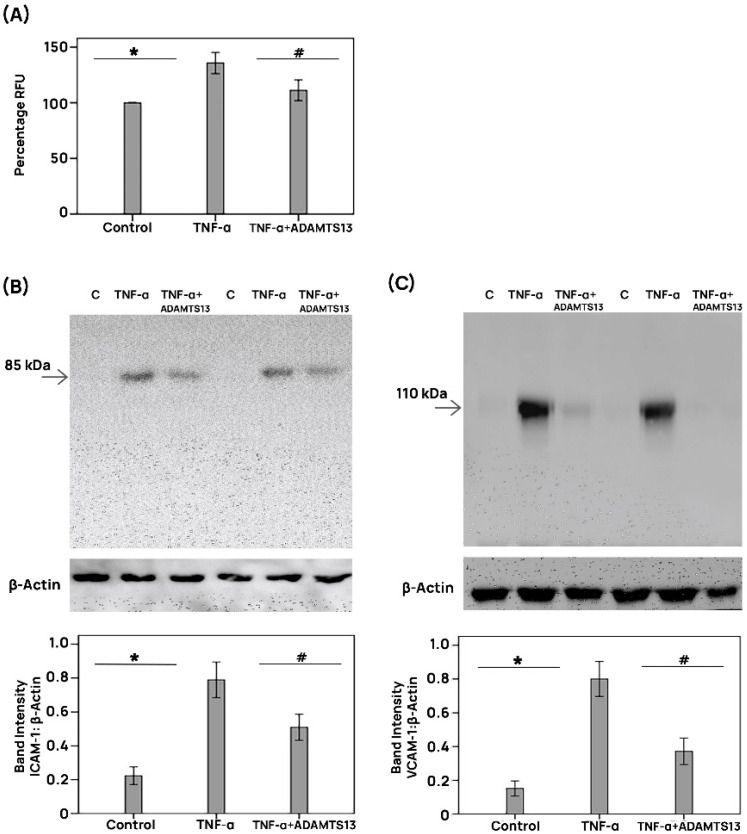
ADAMTS13 affects leukocyte adhesion to human retinal microvascular endothelial cells (HRMECs). HRMECs were left untreated or were stimulated with 1 ng/mL of tumor necrosis factor-α (TNF-α) for 24 h with or without a 1 h preincubation with ADAMTS13 (100 ng/mL). Monocyte adhesion to HRMEC monolayers was assessed with the use of fluorescently labeled THP-1 monocytic cells (**A**). The effects of exogenous ADAMTS13 on protein expression levels of intercellular adhesion molecule-1 (ICAM-1) (**B**) and vascular cell adhesion molecule-1 (VCAM-1) (**C**) were determined with the use of western blots. Results are expressed as means ± standard deviation from three different experiments performed in triplicates. One-way ANOVA and independent t-tests were used for comparisons among three groups and between two groups, respectively. * *p* < 0.05 compared with values obtained from untreated cells. # *p* < 0.05 compared with values obtained from cells treated with TNF-α (RFU = relative fluorescence units).

The adherence of THP-1 monocytes to HRMECs elicited by TNF-α was significantly reduced when HRMECs were pretreated with ADAMTS13 (Figure 9A). This biological effect was substantiated at the molecular level by analysis of ICAM-1 and VCAM levels. Indeed, with the use of western blot analyses, we showed that ADAMTS13 significantly reduced the TNF-α-elicited ICAM-1 (Figure 9B) and VCAM-1 (Figure 9C) levels in HRMECs.

### 3.12. Effect of ADAMTS13 on the Generation of Reactive Oxygen Species in Human Retinal Microvascular Endothelial Cells Cultured in High Glucose

**Figure 10:**
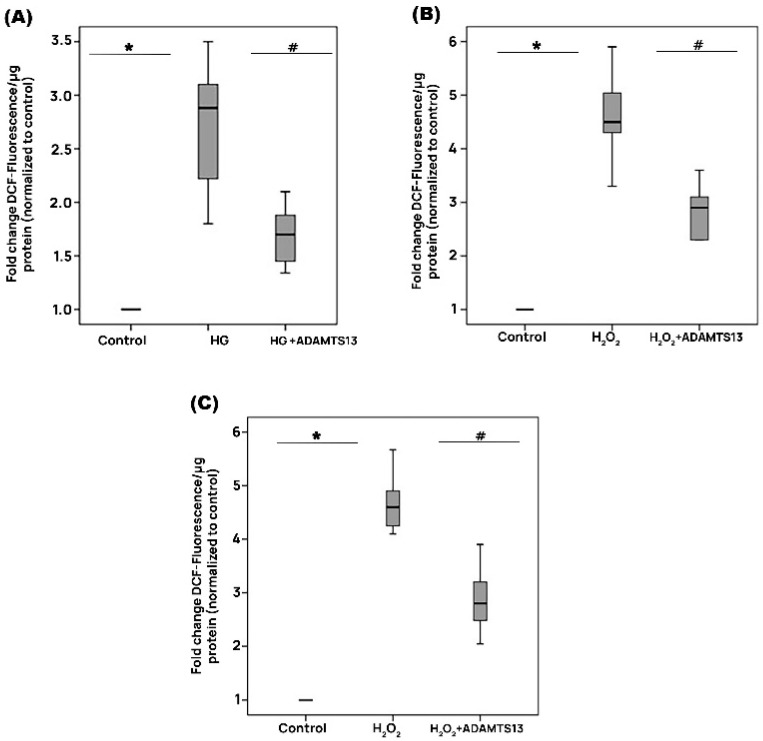
ADAMTS13 reduces cellular oxidative stress. Oxidative stress was induced in HRMECs and human retinal Müller glial cells and monitored with the use of 2′-7′-dichlorofluorescein (DCF) fluorescence intensity analysis. The in vitro effects of cell pretreatment with 100 ng/mL of ADAMTS13 for 1 h were quantified. HRMECs were left untreated (control) or were treated with 25 mM of high glucose (HG) for 24 h. A total of 25 mM of mannitol was used as a control condition for the HG treatment (**A**). Human retinal Müller glial cells (**B**) and HRMECs (**C**) were left untreated or were treated with 10 mM of hydrogen peroxide (H_2_O_2_) for 1 h. Data are provided as medians (interquartile range) from independent (*n* = 3) experiments, each performed in triplicates. Statistical comparisons were performed as described in Section 2.12. * *p* < 0.5 indicates comparisons with values obtained from control cells. # *p* < 0.05 indicates comparisons with values obtained from cells treated with HG or H_2_O_2_.

In response to the HG treatment, ROS generation was highest at an interval time of 24 h. Therefore, the protective effect of ADAMTS13 was examined at this time point. Figure 10A shows the changes in the DCF fluorescence signal, which is a ROS indicator, in HRMECs. HG caused a significant increase in DCF fluorescence compared with that of the control. Pretreatment with ADAMTS13 significantly attenuated ROS generation induced by HG.

### 3.13. Effect of ADAMTS13 on the Generation of Reactive Oxygen Species in Human Retinal Müller Glial Cells and Human Retinal Microvascular Endothelial Cells

The highest ROS generation in response to treatment with the exogenous reactive oxygen species H_2_O_2_ was observed after 1 h. Accordingly, the protective effect of ADAMTS13 was investigated at this time point. Treatment of Müller cells (Figure 10B) and HRMECs (Figure 10C) with H_2_O_2_ induced a significant increase in ROS generation compared to the control. Pretreatment with ADAMTS13 significantly attenuated ROS generation induced by H_2_O_2_ (Figure 10B,C).

### 3.14. Effect of ADAMTS13 on Migration of Human Retinal Microvascular Endothelial Cells

**Figure 11:**
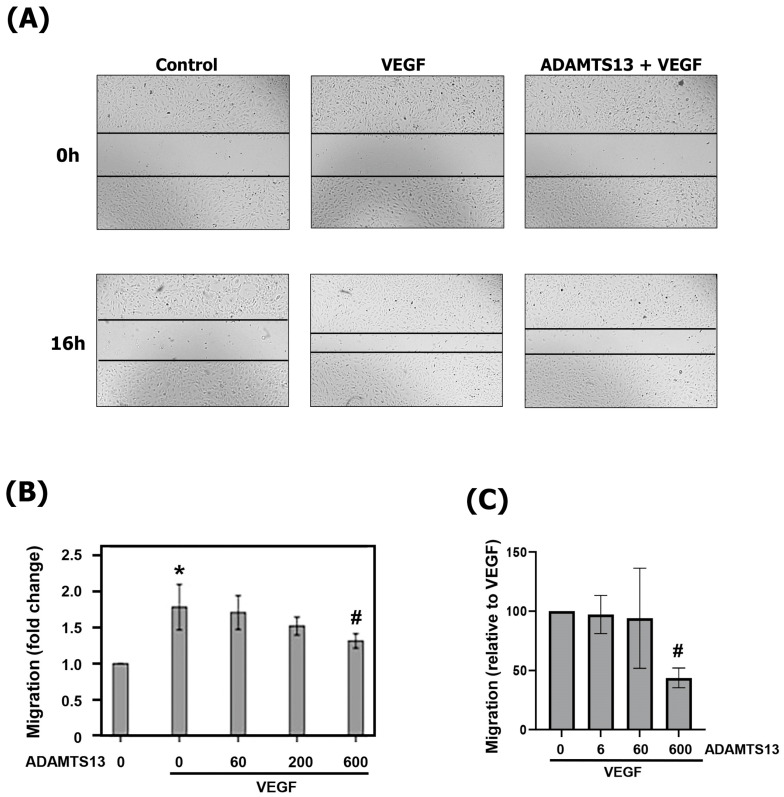
Inhibition of endothelial cell migration by ADAMTS13. (**A**,**B**) Confluent monolayers of overnight starved HRMECs were scratched with sterile micropipette tips and monolayer regeneration was microscopically monitored, subject to various treatments. In one set of experiments, the cultures were pretreated with a dilution medium or ADAMTS13 (60, 200, or 600 ng/mL) for 1 h, followed by stimulation with vascular endothelial growth factor (VEGF) (10 ng/mL) for 16 h. Two independent experiments were performed in duplicates and two-to-three independent field images were taken for the migration analysis with the Image J software (summarized in (**B**)). In panel (**A**), representative images illustrate the effect of ADAMTS13, at a dose of 600 ng/mL, on VEGF-induced cell migration. (**C**) In a second set of experiments, endothelial cell migration through 8 µm pores of polyethylene terephthalate (PET) membranes in response to VEGF (10 ng/mL) with or without pretreatment with ADAMTS13 (6 to 600 ng/mL) was analyzed with the xCELLigence instrument (three or four independent experiments in duplicates). Results are expressed as means ± standard deviation. One-way ANOVA and independent t-tests were used for comparisons among five groups and between two groups, respectively. * *p* < 0.05 compared with values obtained from untreated cells. # *p* < 0.05 compared with values obtained from cells treated with VEGF only.

The migration of endothelial cells is a critical component of the angiogenic process. We tested ADAMTS13 for its ability to block migration of HRMECs (Figure 11). When added as a single stimulus to HRMEC cultures, ADAMTS13 did not significantly affect endothelial cell migration. ADAMTS13 pretreatment at a concentration of 600 ng/mL significantly attenuated the migration of HRMECs after stimulation with VEGF in the scratch wound migration assay (Figure 11A,B) and the transmembrane migration assay (Figure 11C). However, at lower concentrations, no significant effect of ADAMTS13 was observed (Figure 11). Treatment with ADAMTS13 did not affect spontaneous migration.

## 4. Discussion

The key novel findings of this study are that (1) ADAMTS13 was specifically localized in monocytes/macrophages in all the examined epiretinal fibrovascular membranes from patients with PDR and that the components of ADAMTS13/VWF/the platelet marker CD41 axis were significantly upregulated in PDR vitreous fluid, (2) the biomarkers of endothelial cell injury and dysfunction, namely soluble VE-cadherin and soluble syndecan-1, were significantly upregulated in PDR vitreous fluid, (3) treatment with intravitreal rhADAMTS13 restored retinal vascular and BRB integrity in STZ-induced diabetic rats, with this effect associated with the upregulation of VE-cadherin and ß-catenin and the downregulation of VWF, platelet recruitment, and the proinflammatory factors ICAM-1, VCAM-1, and HMGB1, (4) treatment of HRMECs with rhADAMTS13 significantly reduced the elevated levels of the markers of endothelial cell injury and dysfunction and the inflammatory chemokines MCP-1/CCL2 and fractalkine/CX3CL1 in the culture media induced by the studied diabetic mimetic conditions, with this paralleled by a reduction in phospho-ERK1/2 and the adhesion molecules ICAM-1 and VCAM-1, and a decreased binding of human monocytes to HRMECs, (5) treatment of human retinal Müller glial cells with rhADAMTS13 significantly attenuated the increased levels of the inflammatory chemokine MCP-1/CCL2 and the proangiogenic and proinflammatory factor MMP-9 in the culture medium induced by diabetic mimetic conditions, (6) treatment with rhADAMTS13 significantly attenuated ROS generation induced by HG and H_2_O_2_ in Müller glial cells and HRMECs, and (7) treatment with rhADAMTS13 attenuated VEGF-induced HRMECs migration, a crucial step in the angiogenesis cascade. Collectively, our data indicate that rhADAMTS13 reduces diabetes-induced retinal inflammatory vasculopathy and microvascular endothelial cell dysfunction.

Immunohistochemical analyses demonstrated that monocytes/macrophages are the main cellular source of ADAMTS13 in the ocular microenvironment of patients with PDR. The immunoactivity of ADAMTS13 was detected in endothelial cells lining new blood vessels in only five of the 14 studied PDR epiretinal fibrovascular membranes. With the use of western blot analysis, we demonstrate that cultured HRMECs did not express ADAMTS13. However, previous reports have demonstrated that human umbilical vein endothelial cells and human umbilical artery endothelial cells express ADAMTS13 [27], and that the inflammatory cytokines interferon-γ and TNF-α downregulate ADAMTS13 expression in human umbilical vein endothelial cells [28]. Several studies have demonstrated heterogeneity among endothelial cells from different tissues in terms of antigen expression, their response to agonists, and functional activity [29,30,31]. We also demonstrated that ADAMTS13 protein was significantly downregulated in the retinas of rats after four weeks of STZ-induced diabetes. These findings shed some light on the mechanism underlying diabetes-induced retinal inflammatory vasculopathy and microvascular endothelial cell dysfunction. Diabetes-induced retinal inflammation could aggravate retinal damage through the reduction of retinal ADAMTS13.

Previous studies have demonstrated that ADAMTS13 may also be involved in regulating angiogenesis and that ADAMTS13 might be either a proangiogenic or an anti-angiogenic factor, depending on the local microenvironment. In vitro studies have demonstrated that ADAMTS13 induces human umbilical vein endothelial cell proliferation, migration, and tube formation, key steps in the angiogenesis process [32]. Incubation of human umbilical vein endothelial cells with ADAMTS13 increased VEGF expression and enhanced phosphorylation of its receptor VEGF-R2. These findings suggest that ADAMTS13-induced angiogenesis occurs via the VEGF-VEGF-R2 signaling pathway [33]. In contrast, ADAMTS13 reduced VEGF-induced human umbilical vein endothelial cell proliferation, migration, and tube formation via direct interaction between the thrombospondin type 1 domain of ADAMTS13 and VEGF [32]. These findings suggest that ADAMTS13 may have additional roles in vascular biology and might promote angiogenesis, but also exert inhibitory effects when angiogenesis is induced by VEGF. In the current study, we demonstrated that ADAMTS13 reduced VEGF-induced in vitro migration of HRMECs. VEGF is known to be an important player in inducing retinal vascular leakage and angiogenesis in diabetic retinopathy [34].

Vascular endothelial (VE)-cadherin is an endothelial transmembrane glycoprotein with a major role in cell–cell adhesion at the adherence junctions. ß-catenin is a member of the endothelial adherence junction proteins and is a key regulatory component of the VE-cadherin cell adhesion complex. The proteoglycan syndecan-1 is the main component of endothelial glycocalyx which maintains endothelial integrity. When endothelial integrity becomes disrupted during hypoxia and inflammatory conditions, VE-cadherin and syndecan-1 are cleaved and shed into the circulation. Therefore, soluble VE-cadherin and soluble syndecan-1 are considered to be biomarkers reflecting endothelial cell injury [35,36,37,38]. Here, we documented elevated levels of soluble VE-cadherin and soluble syndecan-1 in PDR vitreous fluid, suggesting loss of retinal vascular endothelial cell integrity. We also demonstrated that treatment of cultured HRMECs with rhADAMTS13 significantly reduced diabetic-mimetic-condition-induced elevated levels of soluble VE-cadherin and soluble syndecan-1 in the culture medium. These findings suggest a protective effect of ADAMTS13 on the retinal endothelial barrier function. Indeed, intravitreal injection of rhADAMTS13 improved diabetes-induced downregulation of the adherence junction proteins VE-cadherin and ß-catenin and restored disease parameters of retinal vascular and BRB integrity in STZ- induced diabetic rats. These results complement previous studies showing that rhADAMTS13 provides a protective effect on vascular endothelial barrier function in experimental models of trauma-induced shock [39], intracerebral hemorrhage [40], trauma transfusion [41], and acute kidney injury [12,42].

Severe ADAMTS13 deficiencies are linked to thrombotic thrombocytopenic purpura, characterized by platelet-rich thrombi in the microvasculature [10]. A key role of ADAMTS13 has been identified in downregulating both platelet adhesion and thrombus formation in injured arterioles and in activated venules in an experimental mouse model [43]. rhADAMTS13 reduced cerebral microthrombus formation and brain injury in an experimental mouse model of subarachnoid hemorrhage [17,44]. Here, we documented an increased platelet entrapment within the eyes of patients with PDR by analysis of the platelet biomarker CD41. In line with previous studies, we showed that treatment with intravitreal rhADAMTS13 significantly reduced retinal platelet recruitment in STZ-induced diabetic rats.

In the diabetic retina, prolonged hyperglycemia and altered metabolic pathways increase oxidative stress, upregulate proinflammatory cytokines and chemokines, adhesion molecules, growth factors, and MMP-9, leading to the breakdown of the BRB. Mounting evidence demonstrates a pivotal role of chronic low-grade inflammation in the pathogenesis of diabetic retinopathy. Increased leukostasis has been shown to be a crucial step in the early stages of diabetic retinopathy. In addition, there is abundant evidence for a key role of oxidative stress in the pathogenesis of diabetic retinopathy. Therefore, inhibition of ROS generation in retinal cells could protect the retina from hyperglycemia-induced oxidative damage. Furthermore, a close crosstalk between oxidative stress and inflammation has been established in the pathogenesis of diabetic retinopathy [45,46,47]. In the diabetic retina, inflammatory vasculopathy, endothelial cell dysfunction, and the breakdown of the BRB correlate with an increased adhesion of circulating leukocytes to retinal microvascular endothelial cells [3]. Retinal Müller glial cells and HRMECs are major cell types which are involved in diabetes-induced retinal inflammation [19,21,48]. In the present study, we demonstrated that rhADAMTS13 treatment significantly reduced the expression of the adhesion molecules ICAM-1 and VCAM-1 in cultured HRMECs, decreased the adherence of human monocytic THP-1 cells to these cells, and reduced the production of the inflammatory chemokines MCP-1/CCL2 and fractalkine/CX3CL1 in the culture supernatants of HRMECs induced by the studied diabetic mimetic conditions. In addition, rhADAMTS13 treatment significantly attenuated the production of MMP-9 and MCP-1/CCL2 by Müller glial cells induced by the studied diabetic mimetic conditions. In addition, the intravitreal administration of rhADAMTS13 restored retinal vascular and BRB integrity and normalized the retinal expression of ICAM-1, VCAM-1, the proinflammatory alarmin HMGB1, and phospho-ERK1/2 in STZ-induced diabetic rats, further corroborating the working hypothesis of rhADAMTS13 as a new therapeutic option for the management of diabetic retinopathy. These findings are in line with previous studies showing that rhADAMTS13 provides protection against inflammation in experimental models of renal ischemia–reperfusion injury [12], myocardial ischemia [13], collagen-induced arthritis [15], and diabetic nephropathy [11]. Furthermore, oxidative stress plays a key role in retinal vascular endothelial dysfunction in the diabetic retina [45,49]. Here, we also documented that treatment with rhADAMTS13 attenuated the production of ROS by Müller glial cells and HRMECs following exposure to HG- and H_2_O_2_-induced oxidative stress. Similarly, rhADAMTS13 counteracted oxidative stress in an experimental model of acute kidney injury by cleaving VWF [42].

## 5. Conclusions

In conclusion, our findings suggest that therapeutic approaches to enhance ADAMTS13 levels could potentially be a promising strategy to ameliorate diabetes-induced retinal inflammation and vascular dysfunction. Indeed, recent studies have demonstrated the efficacy and safety of rhADAMTS13 in patients with congenital thrombotic thrombocytopenic purpura [50].

## Data Availability

The data presented in this study are available on request from the corresponding author.
